# Mechanical, Thermal, and Flammability Properties of Eco-Friendly Nanocomposites from Recycled PET/PA-11 Blends Reinforced with Graphene Nanoplatelets

**DOI:** 10.3390/polym17081038

**Published:** 2025-04-11

**Authors:** Unsia Habib, Mohammed E. Ali Mohsin, Zahid Iqbal Khan, Zurina Mohamad, Norhayani Othman, Suleiman Mousa, SK Safdar Hossain, Syed Sadiq Ali

**Affiliations:** 1 Faculty of Chemical and Energy Engineering, Universiti Teknologi Malaysia, Johor Bahru 81310 UTM, Johor, Malaysia; habib20@graduate.utm.my (U.H.); norhayani@utm.my (N.O.); 2 Department of Chemical Engineering, University of Engineering and Technology, Peshawar 25000, Pakistan; 3 Department of Chemical Engineering, College of Engineering, King Faisal University, P.O. Box 400, Al Ahsa 31982, Saudi Arabia; saamousa@kfu.edu.sa (S.M.); snooruddin@kfu.edu.sa (S.S.H.); ssali@kfu.edu.sa (S.S.A.)

**Keywords:** RPET/PA-11, nanocomposites, thermoplastics, graphene nanoplatelets

## Abstract

This study investigates the development of sustainable nanocomposites using recycled polyethylene terephthalate (RPET) and polyamide 11 (PA-11) blends reinforced with graphene nanoplatelets (GNPs). RPET/PA-11 blends were compatibilized with 2 phr Joncryl^®^ and processed using melt blending followed by injection moulding. The effects of varying GNP contents (1–4 phr) on mechanical, thermal, and flame-retardant properties were analysed. The nanocomposite with 1 phr GNPs exhibited an optimal balance of mechanical, flame-retardant, and thermal properties, along with improved dispersion compared to higher GNP loadings. Higher GNP concentrations led to increased stiffness but also promoted agglomeration, which negatively impacted tensile and impact strength. Thermal analysis revealed that GNPs influenced the cold crystallization behaviour of RPET, while the TGA results indicated a moderate enhancement in thermal stability. The maximum degradation temperature (T_max_) increased from 410.38 °C to 430.06 °C with 1 phr GNPs but declined at higher loadings. Similarly, flammability tests showed an improvement in the limiting oxygen index (LOI) from 19 to 24. Morphological analysis confirmed that GNPs facilitated PA-11 dispersion within the RPET matrix, particularly at lower GNP concentrations (1 phr). These findings highlight the potential of RPET/PA-11/GNP nanocomposites for multifunctional applications, providing an optimal balance between mechanical performance, thermal stability, and flame resistance. This research highlights the synergistic effect of GNPs in achieving sustainable, high-performance materials, addressing the challenges of plastic waste management and the need for eco-friendly engineering solutions for industries such as automotive, packaging, and construction.

## 1. Introduction

Polyethylene terephthalate (PET) is one of the most widely used polymers globally, especially in packaging, textiles, and consumer goods, due to its excellent chemical resistance, high rigidity, and thermal stability [1]. However, the massive consumption of PET and its non-biodegradable nature have led to significant environmental concerns, as improper disposal in landfills and water bodies creates persistent plastic pollution [2,3]. Recycling PET into its thermoplastic form (RPET) is a highly attractive approach to mitigate these issues [4,5]. Unfortunately, the mechanical and thermal properties of RPET degrade during recycling due to hydrolytic chain scission and thermo-mechanical breakdown, leading to a decline in molecular weight and melt viscosity [6]. Addressing these limitations requires innovative material development, combining RPET with other polymers or nanofillers to improve its properties [7,8].

Various studies have explored the blending of PET with different polymers, including HDPE and PP [9,10], polystyrene (PS) [11], polycarbonate [12], and polyamide variants, such as PA6 [7] and PA-11 [4,8,13]. Among these, PA6 has been widely examined in combination with RPET [7,14]. However, RPET/PA6 blends often encounter significant drawbacks, including high moisture absorption, reduced impact strength, hydrophilic tendencies, and the degradation of PA6 during prolonged processing. These challenges highlight the necessity for improved blending techniques and innovative filler systems to enhance the properties of RPET blends and broaden their industrial applications [7]. In contrast, PA-11, a bio-based polymer derived from renewable resources, exhibits remarkable mechanical strength, flexibility, and chemical resistance [15]. Blending RPET with PA-11 presents a promising approach to developing a sustainable polymer matrix with enhanced mechanical and thermal properties. However, the inherent immiscibility of RPET and PA-11 leads to phase separation, negatively affecting the structural integrity and performance of the resulting material [16]. Addressing these phase separation issues through compatibilization strategies and advanced nanofiller reinforcement is crucial for realizing high-performance RPET/PA-11 blends suitable for various engineering applications. To overcome these challenges, researchers have explored the use of compatibilizers and chain extenders in PET/PA6 systems, including Poly(isoprene-g-Maleic Anhydride) [14], Joncryl^®^ [17], and copoly(ester-amide 6) [18], among others. Among these, Joncryl^®^ has been widely recognized for its effectiveness in enhancing the properties of PET/PA6 blends, primarily due to its epoxy functional groups, which react with the hydroxyl and carboxyl groups of PET, as well as the terminal amine groups of PA6, facilitating chain extension and improved compatibility [17]. RPET/PA-11, a relatively new blend, was previously studied with Joncryl^®^ (4468) as a compatibilizer and chain extender to enhance interfacial adhesion and phase compatibility [16,19]. While this approach led to some improvements, the resulting material properties were still insufficient for demanding applications, particularly in areas requiring greater thermal stability. Therefore, additional reinforcement strategies are necessary to further enhance the structural integrity and functional performance of RPET/PA-11 blends. However, research on RPET/PA-11 systems has primarily focused on mechanical properties, leaving critical aspects, such as thermal stability, flame resistance, and rheological behaviour, largely unexplored.

Nanofillers, such as graphene nanoplatelets (GNPs), are revolutionizing polymer nanocomposites due to their extraordinary mechanical strength, thermal conductivity, and electrical properties [20]. GNPs are highly attractive for enhancing polymer matrices, offering increased properties in structural health monitoring systems [21,22], barrier properties [23], thermal stability, and reinforcement capabilities [24]. Despite these advantages, achieving uniform dispersion and strong interfacial bonding in polymer systems remains a challenge, particularly at higher GNP loadings [25]. While GNPs have been widely investigated in polymer systems, their integration into RPET/PA-11 blends is unprecedented, particularly concerning their influence on thermal, flame retardant, and rheological properties. The dispersion of GNPs in thermoplastics influences the overall performance of nanocomposites. Achieving even dispersion always remains a challenge due to strong van der Waals forces, leading to agglomeration at higher loadings [26]. Researchers have investigated dispersion techniques that include melt blending, solution mixing, and in situ polymerization, with melt blending being the most technically feasible for thermoplastics [27]. However, moving beyond the optimal GNP dispersion threshold can adversely impact mechanical reinforcement by disrupting stress transfer and altering electrical conductivity due to excessive agglomeration [28]. Therefore, selecting the proper GNP concentration and processing technique is vital for improved properties with minimized agglomeration.

This research marks the first comprehensive exploration of GNP-reinforced RPET/PA-11 nanocomposites, addressing a significant gap in the literature. By leveraging the synergistic effects of GNPs and a compatibilizer (Joncryl^®^), this study not only introduces a novel material system but also expands the scope of RPET/PA-11 blends to include detailed analyses of thermal resistance, flame retardancy, and rheological behaviour. Previous research in this domain primarily focused on mechanical performance and compatibility [4,19], while critical aspects of advanced functionalities essential for applications in automotive, electronics, and sustainable packaging were overlooked.

The novelty of this work lies in introducing graphene nanoplatelets into RPET/PA-11 blends, not only for enhancing some mechanical properties but also for achieving superior thermal and flame resistance. By addressing both environmental and functional challenges, this study contributes to advancing sustainable materials research while providing a foundation for future innovations in nanocomposite development.

## 2. Experimental Section

### 2.1. Materials

Recycled polyethylene terephthalate (RPET) at post-industrial grade was obtained from Alba Polyester Sdn. Bhd., Senai, Malaysia. The properties of RPET supplied by the manufacturer included an intrinsic viscosity of >0.68 dl/g (ASTM D4603-03) [29], an ash content of <0.04% (ASTM D5630-05) [30], crystallinity of at least 25% (DSC), and a density of 1470 kg/m^3^. Polyamide 11 (PA-11), with its code name BMN G8 TLDA, was sourced from Arkema, Philadelphia, PA, USA. The properties supplied by the manufacturer (Alba Polyester Sdn. Bhd., Malaysia) for pure PA-11 included a density of 1030 kg/m^3^ (ISO 1183) [31], a melting temperature of 186 °C, (10 °C/min, ISO 11357-1/-3) [32], an injection moulding melt temperature of 270 °C (ISO 294) [33], and an injection moulding pressure at hold 16 MPa (ISO 294) [33]. Joncryl^®^ ADR 4468 was supplied by BASF Corporation, Ludwigshafen, Germany. The properties of the pure Joncryl^®^ were a specific gravity of 1.08 (25 °C), a molecular weight of 7250, T_g_ (59 °C), a non-volatile content of GC [%] > 99, and an epoxy equivalent weight of [g/mol] 310. Graphene nanoplatelets (GNPs) were procured from Soochow Hengqiu Graphene Technology, Suzhou, China, having the following properties: a bulk density of 0.5–1.0 g/cm^3^ and an ignition temperature of >600 °C. The GNPs had 6–10 monolayers of graphene with average lateral dimensions of ~38 µm and ~13 µm.

### 2.2. Extrusion Processing

The RPET/PA-11/Joncryl^®^/GNP nanocomposites were prepared using a well-defined processing approach. The RPET/PA-11 blend was formulated at an 80:20 wt% ratio, incorporating 2 phr of Joncryl^®^ 4468 as a compatibilizer. The optimization of Joncryl^®^ at this concentration was previously established and documented in our prior study [19]. In the optimized RPET/PA-11/Joncryl^®^ blend, varying concentrations of graphene nanoplatelets (GNPs) (0, 1, 2, 3, and 4 phr) were introduced to assess their influence on the nanocomposite properties. Initially, all raw materials, including RPET, PA-11, Joncryl^®^, and GNPs, were thoroughly mixed physically and subjected to drying in an oven (Memmert, ULM 500, GEMINI LAB, DG Apeldoorn, The Netherlands) at 90 °C for 24 h to eliminate residual moisture prior to processing. The nanocomposites were then produced using the melt blending technique in an extruder (WERNER & PFLEIDERER ZSK25, Stuttgart, Germany), which has a screw diameter of 25 mm and an L/D ratio of 40:1. The extruder is co-rotating and vented, equipped with six heating zones and a screw speed range of approximately 50–500 rpm. For this study, the screw speed was set to 80 rpm, and the feed rate was maintained at 4 kg/h to ensure consistent material flow and effective dispersion of the GNPs. The temperature profile was controlled, starting at 240 °C in the first zone and gradually increasing to 290 °C in the final zone, optimizing the thermal processing conditions for the RPET/PA-11 blend.

### 2.3. Injection Moulding and Formulation of GNP Nanocomposites

Following extrusion, the nanocomposites were shaped into test specimens using an injection moulding machine (NI00B II, JSW PTE LTD, Tokyo, Japan) in accordance with ASTM standards. The injection moulding process was conducted at temperatures between 260 °C and 290 °C from the hopper to the nozzle, with an injection cycle time of 24 s. The injection pressure was set to 80 MPa, while the holding (discharge) pressure was maintained at 60 MPa. The mould temperature was controlled at approximately 60 °C to ensure proper material flow, optimal crystallization, and dimensional stability of the nanocomposites. According to previous studies by Khan et al. [4,19], the tensile strength and flexural strength of rPET were reported to be 18.5 MPa and 27.9 MPa, respectively, with an Izod impact strength of 110 J/m. For PA-11, the ultimate tensile strength was noted as 53 MPa, with an impressive impact strength of 1371 J/m [4]. Table 1 highlights the formulation details of the prepared nanocomposites, where “phr” (parts per hundred resin) indicates the filler amount relative to 100 g of polymer. For instance, a filler content of 1 phr corresponds to 1 g of filler for every 100 g of polymer.

### 2.4. Characterization

The functional groups and structural characteristics of the RPET/PA-11/Joncryl^®^ blend, along with its nanocomposites, were analysed using a PerkinElmer (Waltham, MA, USA) 1600 FTIR spectrophotometer equipped with the attenuated total reflectance (ATR) technique. The spectral acquisition was performed over a wavelength range of 4000–500 cm⁻^1^, with a resolution of 4 cm⁻^1^ and 32 scans per spectrum to improve the signal-to-noise ratio. Fourier transform infrared (FTIR) spectroscopy was employed to investigate the interactions between the RPET/PA-11/Joncryl^®^ matrix and the incorporated GNP nanofillers. The spectral analysis was conducted over a wavelength range of 4000 to 300 cm⁻^1^, with each sample undergoing a detailed scan to ensure precise and consistent results throughout this study. The morphology of the blend having no GNP filler (GNCS-0) was studied through scanning electron microscopy (TM3000, Hitachi High-Tech, Hitachi, Japan). The acceleration voltage was 15 kV with 2.0K magnification under high vacuum. The SEM micrograph was recorded at 30 µm. The morphology of the nanocomposites (GNCS-1 to GNCS-4) was examined using a field emission scanning electron microscope (FESEM, Carl Zeiss Microscopy GmbH, Oberkochen, Germany). Prior to analysis, the samples for SEM and FESEM were coated with platinum using a coating system from Quorum Technologies Ltd., Laughton, East Sussex, UK. The FESEM analysis was performed at an accelerating voltage of 5 kV for GNCS-1 to GNCS-4, while the micrographs were recorded at 2 µm for the nanocomposites with 20K magnifications. The tensile strength of the nanocomposites was evaluated following the ASTM D-638 standard [34], using a pre-load of 0.01 MPa, a test speed of 10 mm/min, and a secant modulus preset at 1%. Mechanical properties, including tensile and flexural strengths, were analysed using a Zwick/Roell (Ulm, Germany) Z020 universal testing machine. Flexural tests were conducted in accordance with ASTM D790 [35], employing a cross-head speed of 1 mm/min and a pre-load of 0.1 MPa. The test was performed with a support span of 100 mm. A 20 kN load cell was used for both tensile and flexural assessments. The flexural test samples measured 127 mm in length, 12.3 mm in width, and 5 mm in thickness. Impact testing was carried out using a Zwick Roell/HIT25P machine, adhering to ASTM D256-10, using a pendulum energy of 11 Joules. Each formulation was tested at least five times under room temperature conditions to ensure reliability. The specimens were conditioned for mechanical testing at 23 °C (±2 °C) and 50% (±10%) RH for 48 h, in accordance with ASTM D638 and ASTM D618 standards, before testing. This conditioning ensures moisture equilibrium, minimizing environmental influences on mechanical properties.

The thermal behaviour, including the glass transition temperature (Tg) and melting temperature (Tm), was analysed using a Perkin-Elmer Differential Scanning Calorimeter (DSC). The samples were heated from ambient temperature to 300 °C and subsequently cooled to room temperature, all under an inert nitrogen atmosphere to prevent oxidation and other environmental effects. The temperature ramp rate was maintained at 10 °C per minute. Thermogravimetric analysis (TGA) was conducted to examine the thermal properties of the RPET/PA-11/Joncryl^®^ blend, nanocomposites, and hybrid nanocomposites. The analysis was performed using a Perkin TGA 7, USA. Approximately 5 mg of each sample was placed under a nitrogen atmosphere to prevent oxidation during testing. The temperature was increased at a rate of 10 °C per minute over a range of 30 °C to 900 °C, with a nitrogen flow rate maintained at 50 mL/min. This procedure provided insights into the thermal stability and degradation behaviour of the materials.

The LOI test was used to determine the minimum concentration of oxygen required to ignite and sustain the combustion of the samples. The testing followed the ASTM D2863-2013 standard procedure and was performed using an LOI device from Rheometer Scientific, UK. Five samples for each formulation, with dimensions of 100 × 10 × 10 mm^3^ (length × width × thickness), were prepared. The tests were conducted under varying oxygen and nitrogen (O₂/N₂) environments to determine the precise O₂/N₂ ratio needed to burn 5 cm of the sample within 3 min. The average LOI value for each formulation was recorded based on the results from five replicates [36]. These values were analysed to assess the flammability characteristics of the materials. The UL-94 test was performed to evaluate the material’s reaction to a fire hazard and to measure the time taken for the flame to self-extinguish. This test was carried out using a manually adjusted UL-94 chamber in compliance with ASTM D3801 standards. Five samples, each measuring 130 × 13 × 3 mm^3^ (length × width × thickness), were tested. The results of the UL-94 analysis were categorized as V-0, V-1, or V-2 based on the burning ratings [37].

## 3. Results and Discussion

### 3.1. FTIR Analysis of GNP-Filled RPET/PA-11 Nanocomposites

**Figure 1:**
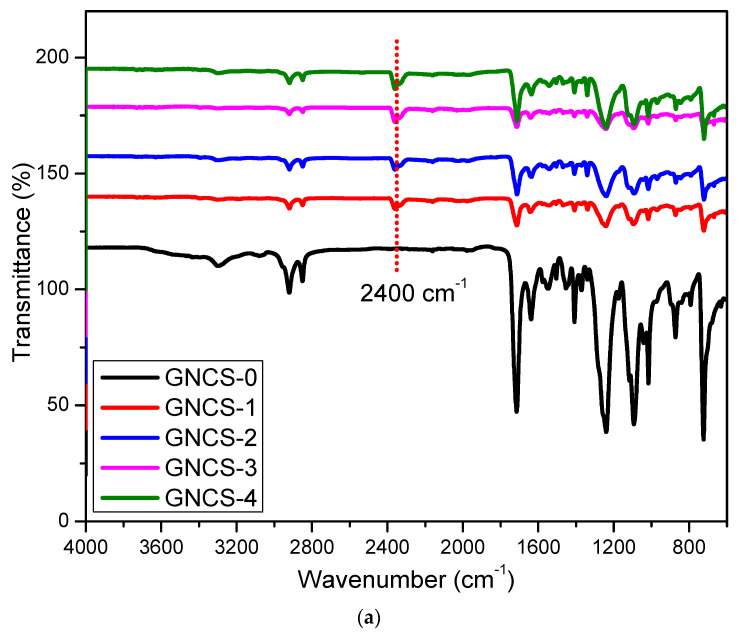

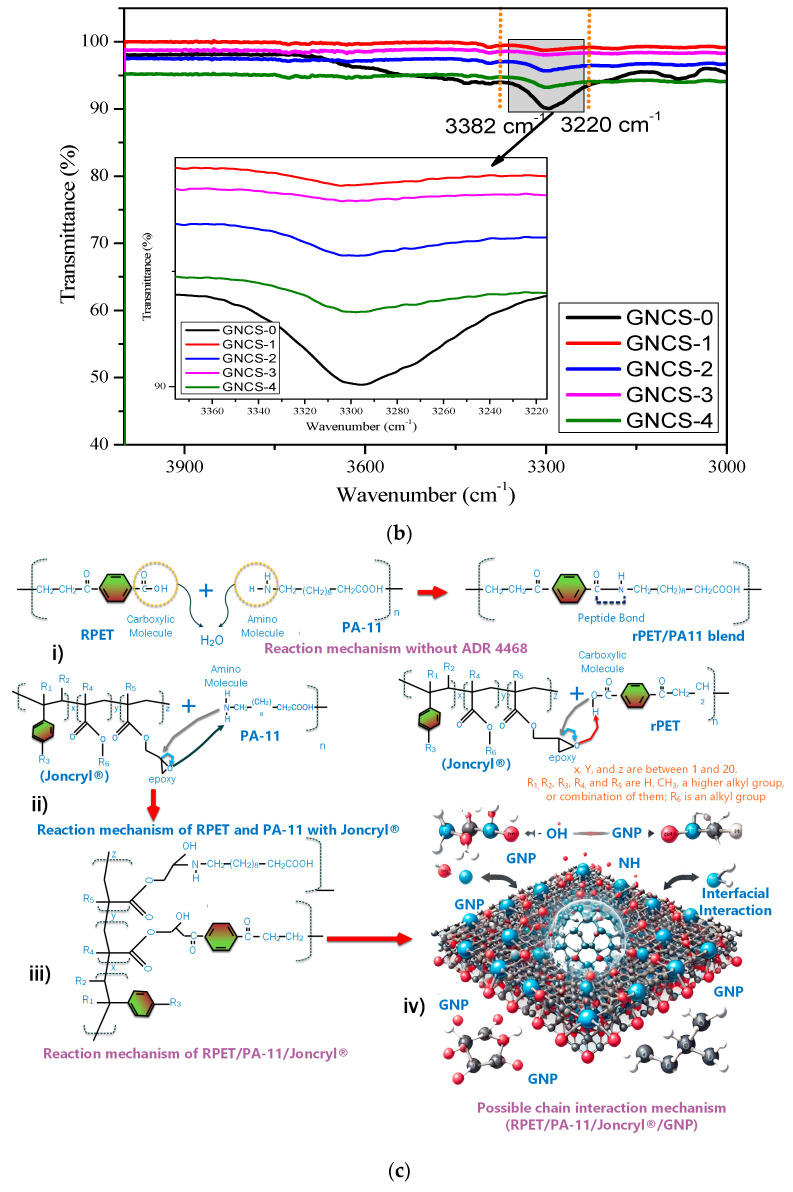
(**a**) FTIR spectra of GNP nanocomposites (note, to improve clarity and avoid overlapping, transmittance values are offset vertically by +20 for GNCS−0, +40 for GNCS−1, +60 for GNCS−2, +80 for GNCS−3, and +100 for GNCS−4). (**b**) FTIR zoom spectra without offset. (**c**) Possible chain interaction mechanisms.

**Figure 2:**
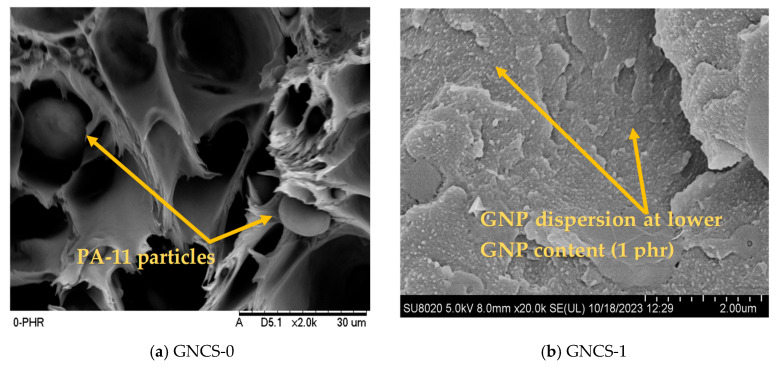

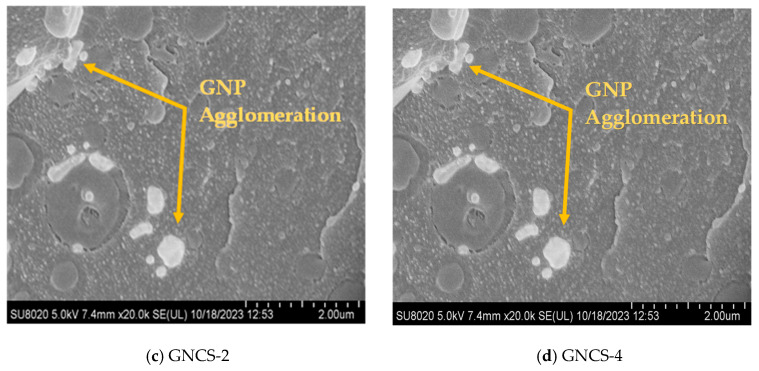
FESEM micrographs of (**a**) GNCS-0, (**b**) GNCS-1, (**c**) GNCS-2, and (**d**) GNCS-4.

The structural modifications and interactions within the GNP nanocomposites containing different GNP concentrations (0–4 phr) were examined using Fourier transform infrared (FTIR) spectroscopy, as illustrated in Figure 1a,b. The spectra displayed absorption signals within the 3220 to 3382 cm⁻^1^ range (Figure 1b), indicating the presence of -NH and -OH groups, particularly in the GNCS-0 blend having 0 phr of GNPs. A distinct absorption peak around 2400 cm⁻^1^ is observed in GNCS-0 (Figure 1a) but is absent in GNP-containing samples, potentially indicating differences in adsorbed atmospheric CO₂ or interfacial interactions. The 2025 study explained the absence of the 2400 cm^−1^ peak with the addition of GNPs in an HDPE matrix [38]. In GNP/HDPE nanocomposites, the absence of the ~2400 cm⁻^1^ peak (present in neat HDPE but not in GNP-containing samples) correlates with enhanced interfacial bonding and barrier properties [38]. Additionally, the carbonyl region (~1700 cm⁻^1^) remains unchanged across all formulations, suggesting that GNPs primarily enhance physical interactions through hydrogen bonding and dispersion effects, rather than forming strong covalent bonds with the polymer matrix. This aligns with morphological analysis (Figure 2), where GNP incorporation improved the dispersion stability of PA-11 particle (Figure 2b) and mitigated the coalescence of PA-11, which is visible in Figure 2a (GNCS-0).

In the GNP-containing nanocomposites, the spectral changes reflected interactions between GNP and the matrix. Graphene is considered to promote chain interaction. As a result, the peak intensity within the 3281 to 3405 cm⁻^1^ range in the nanocomposites decreased, suggesting the involvement of -OH groups in additional physical bonding interactions. This trend of peak reduction, indicating enhanced molecular interactions, aligns with previous research findings [16]. The affinity between GNP nanofillers and the compatibilized blend (GNCS-0) is likely due to partial polarity compatibility, facilitating better interfacial interactions. While pristine GNPs are generally considered hydrophobic, prolonged exposure to ambient conditions can lead to partial surface oxidation, introducing hydroxyl (-OH) and carboxyl (-COOH) functional groups, as supported by the literature [39,40]. These oxygen-containing groups enhance the hydrophilicity of GNPs, improving their interaction with the polymer matrix through hydrogen bonding and better dispersion. Additionally, during high-temperature melt blending (~240–290 °C), minor oxidative effects may further modify the GNP surface, improving interfacial adhesion within the nanocomposite. The observed reduction in FTIR peaks around 3200–3400 cm⁻^1^ suggests stronger hydrogen bonding interactions, which aligns with previous findings where similar peak shifts confirmed improved polymer–filler interactions [38,41]. Furthermore, the uniform dispersion and distribution of GNPs, as illustrated in Figure 2b, further validate the effective interaction among all components within the nanocomposite. However, distinguishing these interactions in the FTIR spectra remains challenging due to the overlapping -NH and -OH groups. The FTIR spectra (Figure 1a,b) indicate variations in the intensity of the -OH and -NH absorption peaks with GNP incorporation. While a general reduction in peak intensity is observed, the trend is not strictly linear across all formulations. This fluctuation can be attributed to differences in GNP dispersion, hydrogen bonding interactions, and possible structural rearrangements within the polymer network. The presence of GNPs likely disrupts some hydrogen bonds between RPET and PA-11 while also forming new interfacial interactions, leading to localized variations in peak intensity. Irregularities in FTIR peak behaviour due to nanofiller–polymer interactions have been reported in previous studies [42]. This enhanced interaction at reduced GNP phr (GNCS-1) plays a vital role in improving the mechanical performance of the nanocomposites. The morphological result also supports the same phenomena as small aggregates of GNPs are visible in the GNP nanocomposites (Figure 2c,d) beyond 1 phr of GNP content. At 1 to 4 phr GNPs (GNCS-1 to GNCS-4), the FTIR spectrum displayed reductions in peak intensity and broadening in the 3282–3400 cm⁻^1^ range, suggesting moderate hydrogen bonding between GNPs’ oxygen-containing groups and the matrix’s amide groups; similar results were reported by other researchers [43]. GNPs tend to aggregate due to van der Waals forces and π−π interactions between graphene layers, which makes their dispersion in polymer matrices challenging. The same phenomenon was also reported by Awwad et al. (2021) [44].

Beyond the loading of 1 phr GNPs, the reduced efficiency of interfacial bonding, as evidenced by the increased peak intensity for the 2 to 4 phr GNP nanocomposites (GNCS-2 to GNCS-4) as compared to the 1 phr GNP nanocomposites (GNCS-1). The same correlated with declining mechanical performance and the onset of GNP agglomeration. Similar observations have been reported by researchers for 3D printing, where agglomerated GNPs disrupted the uniform bonding between the printed layers and created weak points that compromised the structural integrity of the nanocomposites [45].

A proposed chain interaction mechanism is illustrated in Figure 1c, detailing the key interactions within the nanocomposite system, where (i) depicts the reaction between the carboxyl (-COOH) group of RPET and the amine (-NH₂) group of PA-11, forming a peptide bond, with H₂O generated as a byproduct [4]; (ii) represents the interaction between Joncryl^®^’s epoxy groups and the carboxyl groups of RPET, as well as the amine groups of PA-11, facilitating improved compatibilization; (iii) demonstrates the chain extension effect of Joncryl^®^ on the RPET/PA-11 matrix [19]; and (iv) illustrates the potential interactions between GNPs and the compatibilized RPET/PA-11/Joncryl^®^ blend, highlighting possible interfacial adhesion mechanisms.

### 3.2. Morphology of GNP Nanocomposites

Figure 2 shows SEM and FESEM micrographs of the tensile fracture samples for the RPET/PA-11/Joncryl^®^/GNP nanocomposites with 0, 1, 2, and 4 phr GNPs at 30 µm for GNCS-0 blend and 2 µm for GNCS-1 to GNCS-4. The difference in the sizes of the micrograph of nanocomposites with the blend having 0 phr GNPs was due to the size of the GNP nanofillers as the GNP nanofillers were not visible at 30 µm, while the morphology of the 0 GNP blend was not clear at 2 µm. The SEM and FESEM micrographs for GNCS-0, GNCS-1, GNCS-2, and GNCS-4 reveal the morphological features and GNP dispersion in the RPET/PA-11/Joncryl^®^ blends. The analysis emphasizes how the addition of GNPs affects the microstructure and the resulting reinforcement. The control sample (0 phr GNPs) exhibited a fibrous structure, with no visible fillers, as expected for a polymer blend without reinforcement. The lack of reinforcement contributed to a structure with no restriction on polymer chain movement. The presence of GNPs in the RPET/PA-11/Joncryl^®^ matrix significantly influences the phase morphology. In Figure 2a, larger PA-11 particles are observed, indicating limited phase stabilization. However, the addition of 1 phr GNPs contributed to reducing the coalescence of PA-11 droplets, as observed in Figure 2a, where larger PA-11 particles are visible in the absence of GNPs. With the addition of 1 phr GNPs, the PA-11 phase exhibits a more refined and dispersed morphology (Figure 2b), indicating that GNPs act as a physical barrier, preventing droplet growth through coalescence. At 1 phr GNPs, the micrograph showed a uniformly dispersed filler within the polymer matrix. The GNP fillers were well integrated with no apparent signs of agglomeration, resulting in good interfacial interactions.

**Figure 3:**
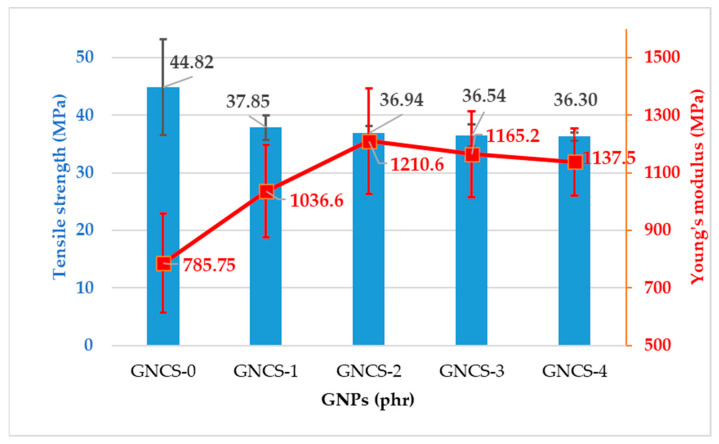
Tensile strength and Young’s modulus of the GNP nanocomposites.

For the 2-phr-GNP nanocomposite, the micrograph showed slightly larger filler domains compared to 1 phr, with minor agglomeration occurring in localized regions. While the GNP fillers were still relatively well dispersed, these clusters began to disrupt the uniformity of the matrix and reduce the effectiveness of the reinforcement. This was consistent with a slight decrease in mechanical properties (Figure 3) observed at this filler loading compared to 1 phr. At 4 phr GNPs, the micrograph showed a clear agglomeration of GNP particles. The clustered filler disrupted the matrix continuity, reduced the interfacial area available for stress transfer, and acted as a stress concentrator. Additionally, Figure 2d highlights the localization of GNPs at the interfacial region, which may contribute to a reduction in interfacial tension, promoting improved phase compatibility. The presence of GNPs acts as a steric hindrance, preventing the aggregation of dispersed PA-11 domains during melt blending and leading to a more stable and refined morphology. These effects collectively enhance the structural integrity of the nanocomposite. Researchers demonstrated GNP agglomeration at higher loading in sugar palm starch (TPS) thermoplastic nanocomposites reinforced with graphene nanoplatelets [46]. Overall, the morphological analysis revealed that 1 phr GNPs had the most uniform dispersion and strongest interfacial binding, resulting in better properties.

### 3.3. Mechanical Properties of GNP Nanocomposites

Figure 3 illustrates the tensile strength and Young’s modulus of the RPET/PA-11/Joncryl^®^/GNP nanocomposites with varying GNP contents (0, 1, 2, 3, and 4 phr). The tensile strength and strain results of the RPET/PA-11/Joncryl^®^/GNP nanocomposites revealed a slight reduction in mechanical properties compared to the control sample (GNCS-0). The performance of the control sample was attributed to the absence of filler-related interference, allowing uninterrupted continuity of the polymer chains. With the addition of 1 phr GNPs (GNCS-1), the tensile strength decreased to 37.85 ± 2.13 MPa, reflecting the influence of the filler on the matrix.

As the GNP content increased from 2 phr to 4 phr, the tensile strength was nearly the same with a minor reduction recorded as 36.94 ± 1.21 MPa and 36.3 ± 0.66 MPa, respectively. The slight decrease in tensile strength was attributed to the onset of GNP agglomeration, as observed in the morphological analysis (Figure 2c,d). These clusters disrupted the uniformity of the matrix, reducing effective stress transfer and weakening the composite slightly. The reduction in tensile strength and strain compared to the control sample was primarily due to the disruption of polymer chain continuity by the filler, the stiffening effect of GNPs that restricted chain mobility, and the increasing agglomeration of GNPs at higher loadings. The overall mechanical properties are listed in Table 2. The tensile results are consistent with the results of Rahmat et al. (2024) that were reported for GNP-based nanocomposites with different thermoplastic polymer matrices [46].

The Young’s modulus results, as shown in Figure 3, demonstrated an increase with the addition of GNPs compared to the control sample (GNCS-0), which exhibited the lowest modulus of 785.75 ± 171.5 MPa. The lower stiffness of the control sample was attributed to the lack of reinforcement since there were no fillers to limit the deformation of the polymer chains. At 1 phr GNPs (GNCS-1), the modulus increased significantly to 1036.6 ± 159.9 MPa, reflecting increased stiffness due to the uniform distribution of GNPs within the polymer matrix. The morphological analysis (Figure 2b) confirmed the uniform distribution of GNPs, which contributed to interfacial bonding and effective load transfer. At 2 phr GNPs (GNCS-2), Young’s modulus further increased to 1210.6 ± 183.3 MPa, the highest value observed among the composites. This indicated that the GNPs effectively increase the stiffness of the matrix due to better dispersion and interaction. The FESEM analysis (Figure 2b) indicated some degree of agglomeration at 2 phr GNPs; the mechanical properties improvements observed at this loading are attributed to the combined effects of dispersed and partially agglomerated GNP networks. Even with minor aggregation, the well-dispersed portion of GNPs enhances stress transfer, restricts polymer chain mobility, and increases stiffness, leading to the observed improvements in Young’s modulus and flexural modulus. This aligns with previous studies where GNPs, even in partially agglomerated states, contributed to reinforcing effects due to their high aspect ratio and strong interfacial interactions with the polymer matrix [47]. However, beyond 2 phr, excessive aggregation disrupts homogeneity, acting as a stress concentrator and reducing the overall reinforcement efficiency. These findings confirm that GNP reinforcement is not solely dependent on perfect dispersion, but, rather, it also depends on an optimal balance between well-dispersed regions and percolated filler networks that contribute to stiffness enhancement. When the GNP content increased to 3 phr (GNCS-3) and 4 phr (GNCS-4), the modulus slightly decreased to 1165.2 ± 148.6 MPa and 1137.5 ± 115.32 MPa, respectively. The reduction in stiffness was attributed to the onset of agglomeration, as indicated in the morphological results (Figure 2c,d), where GNP clustering reduced the effective filler–matrix interface, slightly reducing the reinforcement effect. The aggregation of GNPs can cause weak interfacial adhesion with the polymer matrix, which produces stress raisers inside the matrix [44].

The comparison of Young’s modulus versus impact in Table 2 reveals that the impact strength of the GNCS-1 nanocomposites was nearly the same as the control sample (GNCS-0). Although the addition of GNPs increased stiffness, it maintained the ability of the matrix to dissipate impact energy. At 1 phr GNPs, the composite reached a favourable equilibrium with an improved modulus (1036.6 ± 159.9 MPa) and a moderate impact strength of 221.0225 ± 52.08 MPa. This was attributed to the uniform dispersion of the GNPs, which provided reinforcement without significantly affecting the flexibility of the matrix. At 2 phr GNPs, although the modulus reached a peak value of 1210.6 MPa, the impact strength decreased due to the increased stiffness of the composite. At 3 phr and 4 phr GNPs, the combined effects of agglomeration and reduced flexibility further reduced the impact strength, highlighting a trade-off between stiffness and energy absorption. The trends observed in Young’s modulus and the impact strength aligned with the findings of the tensile properties, thermal analysis, and morphology. The same phenomenon is supported by other researchers who found that uniform dispersion is critical for achieving optimal mechanical performance and that agglomeration at higher concentrations can reduce the reinforcement effect [48].

**Figure 4:**
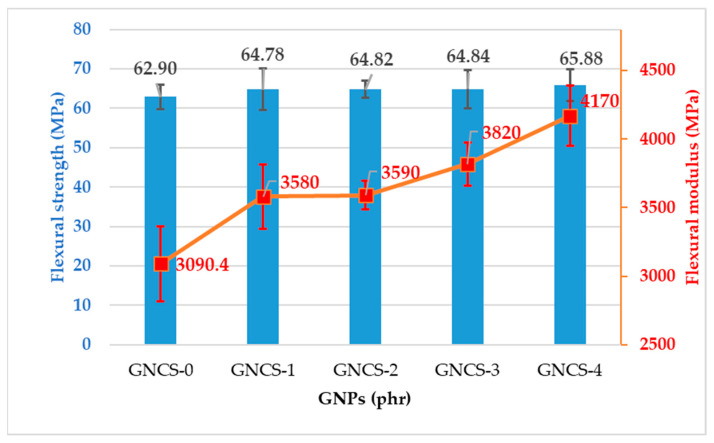
Flexural strength and flexural modulus of the GNP nanocomposites.

The flexural strength and modulus of the GNP nanocomposites are presented in Figure 4. The flexural strength of the control sample was recorded as 62.9 ± 3.10 MPa. At 1 phr GNPs (GNCS-1), the flexural strength increased to 64.78 ± 5.26 MPa. The flexural strength was nearly the same for all the nanocomposites with a slight variation. The difference in the trends of the tensile and flexural strength attributed to the different modes of testing forces and the same phenomena was reported by other researchers [4]. The slight improvement in flexural strength aligns with studies from the literature, which emphasize the role of graphene-based fillers in enhancing flexural strength due to their high stiffness and surface area, enabling better matrix interaction [49]. This trend suggests that matrix reinforcement was maintained up to these loadings with minimal disruption. The mechanism of slight agglomeration creating stress concentration zones is supported by research indicating that while uniform dispersion is ideal, some level of GNP clustering can paradoxically enhance certain mechanical properties under specific conditions [50].

**Figure 5:**
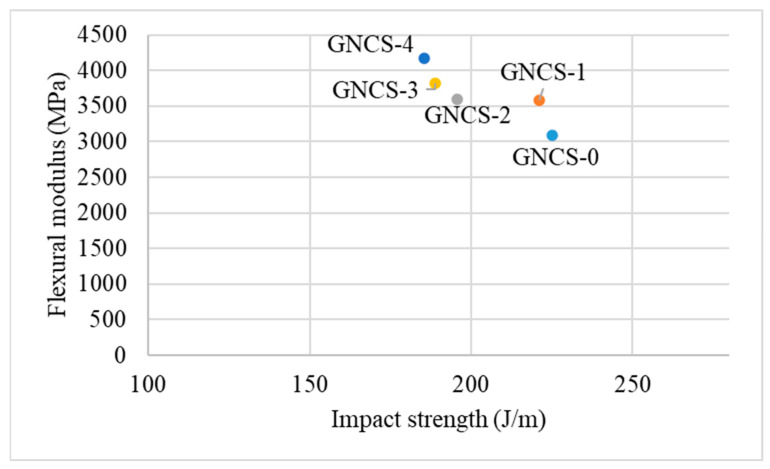
Flexural modulus vs. impact strength of the GNP nanocomposites.

The flexural modulus, as shown in Figure 4, demonstrated a consistent increase with the addition of GNPs compared to the control sample (GNCS-0), while Figure 5 shows the inverse relationship between modulus and impact strength. The flexural modulus of the composites increased steadily with the GNP content, highlighting the stiffening effect of the filler. The control sample (GNCS-0) had the lowest flexural modulus of 3090.4 ± 273.4 MPa. The flexural modulus increased to 3580 ± 237.06 MPa with the addition of 1 phr GNPs. At 3 and 4 phr GNPs (GNCS-3, GNCS-4), the modulus further increased to 3820 ± 157.3 MPa and 4170 ± 217.79 MPa, which was likely due to the increased filler content and aggregation of GNP particles that created localized rigid zones within the matrix. The same phenomenon was reported by other researchers in their study [50].

Figure 5 illustrates the relationship between the flexural modulus and impact strength. The flexural modulus increased with the GNP content, while the impact strength continuously decreased due to the stiffening effect of the filler. At 1 phr GNPs (GNCS-1), the modulus increased with nearly the same impact strength as compared to the control sample (GNCS-0), reflecting the balanced effect of well-dispersed GNPs. However, as the GNP content increased to 2 phr and above, the impact strength decreased. This reduction in impact strength was attributed to the increased stiffness of the matrix and the onset of agglomeration at higher filler loadings, as observed in the morphological analysis (Figure 2d). The agglomerated GNP clusters acted as stress concentrators, further reducing the composite’s ability to absorb and dissipate energy during impact.

The individual mechanical properties vary across the formulations. GNCS-1 demonstrates a well-balanced combination of tensile strength, impact resistance, and modulus, making it the most structurally efficient formulation. Although GNCS-2 has the highest Young’s modulus and GNCS-4 has the best flexural modulus, excessive stiffness in higher GNP loadings can lead to embrittlement, reducing the overall mechanical stability. The FESEM analysis further supports this by showing improved morphology and dispersion at 1 phr GNPs, minimizing phase coalescence and optimizing load transfer. Therefore, GNCS-1 is identified as the most effective formulation for balancing mechanical performance and structural integrity. However, at higher GNP loadings (2–4 phr), the GNP agglomeration (Figure 2c,d) leads to stress concentration points, negatively affecting mechanical properties. However, the mechanical properties of the developed nanocomposites exhibit a significant enhancement compared to those of the RPET and RPET/PA-11 blends. In previous studies, RPET alone demonstrated a tensile strength of 18.5 MPa, a flexural strength of 27.9 MPa, and an impact strength of 110.53 J/m. Meanwhile, an RPET/PA-11 blend (80 wt% RPET, 20 wt% PA-11) exhibited a tensile strength of 32.2 MPa, a flexural strength of 47.3 MPa, and an impact strength of 147.12 J/m [4]. These comparisons highlight the substantial improvements achieved through compatibilization and GNPs, demonstrating the superior mechanical performance of the developed nanocomposites. Furthermore, considering the high cost of GNPs, increasing the filler content beyond 1 phr does not provide substantial benefits in terms of mechanical reinforcement, making GNCS-1 the most efficient and cost-effective formulation.

### 3.4. Differential Scanning Calorimetry (DSC)

**Figure 6:**
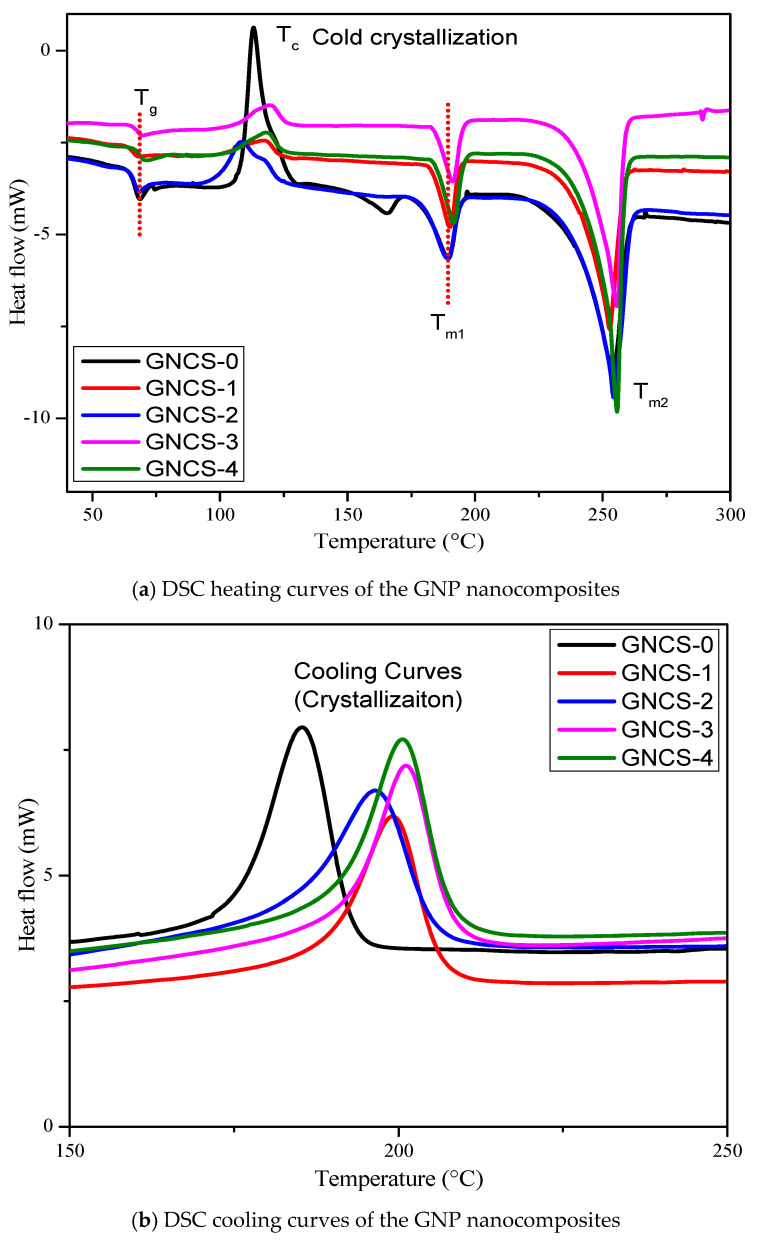
(**a**) DSC heating curves and (**b**) DSC cooling curves of the GNP nanocomposite.

The DSC results are demonstrated in Figure 6a,b and Table 3. The observed T_g_ at ~64 °C in the RPET/PA-11 nanocomposites falls between the T_g_ values of pure RPET (~70–80 °C) [6] and PA-11 (~50–55 °C). This shift suggests potential interfacial interactions between the two phases, influenced by Joncryl^®^ compatibilization and the presence of GNP fillers. Although RPET and PA-11 are considered immiscible, the incorporation of compatibilizers and nanofillers can lead to localized interactions at the phase boundary, restricting polymer chain mobility and shifting T_g_ toward an intermediate value. Similar behaviour has been observed in previous studies on immiscible polymer blends where interfacial modifications alter thermal transitions [51]. These findings indicate that while complete miscibility is not achieved, phase interactions play a role in modifying the thermal response of the nanocomposites. The results in Figure 6a and Table 3 reveal that the glass transition temperature (T_g_) remained within a narrow range (64.45–66.41 °C), indicating that the addition of GNPs has minimal influence on T_g_. However, a slight increase in T_g_ is observed with increasing GNP content. The nanocomposites with 4 phr GNPs (GNCS-4) showed the highest T_g_ (66.41 °C). This suggests that GNPs restrict polymer chain mobility, leading to a more rigid structure.

In the exothermic DSC analysis, during heating, as the temperature increases from 30 °C to 300 °C, certain polymers, particularly recycled PET, exhibit a cold crystallization (Tc) peak appearing in an upward direction at around 110 °C (Figure 6a). Tc exhibits a nonlinear trend with increasing GNP content, indicating a balance between nucleation effects and polymer chain mobility restrictions. The Tc of GNCS-0 appears at 113.19 °C, increasing to 117.08 °C at 1 phr GNPs (GNCS-1), suggesting that GNPs facilitate nucleation and accelerate crystallization. However, at 2 phr GNPs (GNCS-2), Tc drops to 108.41 °C, which may be attributed to stronger interfacial interactions hindering polymer chain rearrangement. As GNP loading increases to 3 phr (GNCS-3), Tc rises significantly to 119.44 °C, indicating enhanced heterogeneous nucleation. A slight reduction at 4 phr GNPs (GNCS-4) to 118.08 °C suggests that excessive GNP loading may lead to agglomeration, reducing nucleation efficiency. These observations align with the crystallinity (*Xc*) trends and mechanical properties, demonstrating that GNP loading significantly influences both thermal and structural performance, with nucleation effects most pronounced at 3 phr, whereas excessive loading may hinder crystallization efficiency. Tc is followed by a cold crystallization melting peak (T_m1_), which occurs as a downward transition in the thermogram, as shown in Figure 6a. Upon further heating, the final melting temperature of the nanocomposites (T_m2_) is observed. A similar cold crystallization phenomenon has been observed in poly(lactic acid) (PLA)/poly(ethylene oxide) (PEO) blends and PLA/PEO nanocomposites incorporating carbon nanotubes (CNTs), as reported in previous studies [52]. Tc displays some deviation, with a slight increase in Tc for GNCS-1, GNCS-3, and GNCS-4, indicating that GNPs act as a nucleating agent, promoting crystallization. However, GNCS-2 shows the lowest Tc (108.41 °C), which might indicate some irregular crystallization process at this nanocomposite, potentially due to GNP dispersion challenges, or it might be due to variations in polymer–filler interactions.

However, the melting temperatures (T_m1_ and T_m2_) remained nearly consistent across all the nanocomposites (Figure 6 and Table 2), confirming that the GNP addition does not substantially change the crystalline phase stability of the RPET/PA-11 matrix. The slight increase in T_m2_ for GNCS-3 (255.13 °C) and GNCS-4 (255.14 °C) compared to GNCS-0 (253.53 °C) signifies that the T_m2_ values of the nanocomposites exhibit a slight increase with GNP incorporation, which suggests minor modifications in the crystalline structure due to filler-induced nucleation effects. However, it is important to note that an increase in T_m2_ alone does not directly indicate improved thermal stability, as the melting temperature primarily reflects the crystalline structure rather than degradation resistance. Instead, thermal stability is better evaluated through TGA analysis, where the T_onset_ and T_max_ values provide clearer evidence of enhanced thermal resistance in GNP-reinforced nanocomposites. The observed slight increase in T_m2_ likely results from restricted polymer chain mobility due to GNP interactions, leading to a more thermally stable crystalline phase but not necessarily higher degradation resistance. The melting temperature (Tm_2_) of GNCS-0 at 0 phr GNPs was 253.53 °C. The Tm_2_ value of the nanocomposites with 1, 2, 3, and 4 phr GNPs was noted at 252.26 °C, 253.66 °C, 255.13 °C, and 255.14 °C, respectively. There was no considerable variation in the Tm_2_ values of the GNP nanocomposites, as displayed in Table 3. The amount of GNPs did not influence the Tm_2_ of the nanocomposites. Markedly, the processing temperature can be maintained if a higher GNP content is incorporated into the nanocomposites.

The degree of crystallinity (*X_c_*) is a key factor affecting the mechanical properties of polymer composites. The crystallinity of all the nanocomposites listed in Table 2 was determined from the crystallization peak in the DSC cooling curve (Figure 6b) using the following equation:

(1) $$Xc\left(\%\right)=\frac{\Delta H}{\Delta Ho\times 0.8}\times 100$$

where Δ*H* represents the normalized melting enthalpy obtained from the crystallization peak of the DSC cooling curve. The parameter Δ*H_o_* corresponds to the heat of fusion for fully crystalline PET, reported as 140 J/g in the literature [53]. The factor 0.8 accounts for the fraction of RPET in the nanocomposites, as RPET comprises 80 wt% of the total composition. Consequently, the observed variations in crystallinity are primarily attributed to the RPET matrix with the influence of GNPs. The *Xc* percentage displays a substantial increase with GNP incorporation. GNCS-0 (4.19%) has the lowest crystallinity, which confirms that the neat RPET/PA-11 blend has poor crystalline order. The significant increase in crystallinity with the addition of GNPs demonstrates the nucleating effect in the RPET/PA-11/Joncryl^®^ blend. In Figure 6b, the crystallization temperature of the cooling curves of the nanocomposites varies depending on the GNP content, indicating that the GNPs act as a nucleating agent, promoting earlier crystallization. The observed shift in crystallinity suggests that GNPs facilitate crystallization by enhancing heterogeneous nucleation sites within the polymer matrix. These findings align with previous studies in which high-aspect-ratio nanofillers have been reported to accelerate crystallization in polymer blends [46,47]. The incorporation of GNPs not only influenced the degree of crystallinity but also modified the thermal behaviour, further supporting their role in structural reinforcement. This effect is consistent across different polymer systems and is attributed to the nucleating behaviour of graphene nanoplatelets [46,47].

Overall, higher *Xc* values indicate that GNPs act as a nucleating agent, enhancing crystallization. The slight increases in T_g_ indicate reduced chain mobility due to GNP–polymer interactions. The shifting of Tc towards higher temperature with increasing GNP content indicates reinforced GNPs’ crystallization-promoting effect, which is also proven in Table 3 with crystallinity calculation.

### 3.5. Thermal Gravimetric Analysis (TGA)

**Figure 7:**
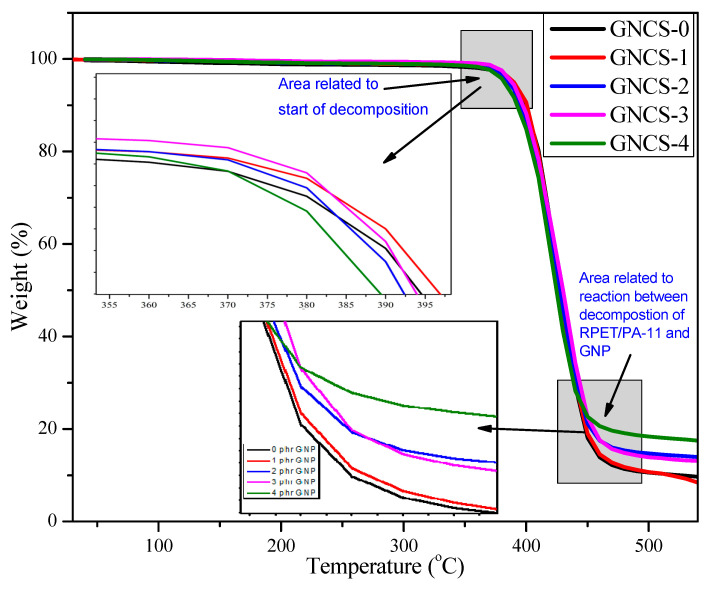
TGA curves of the GNP nanocomposites.

Figure 7 shows the thermogravimetric analysis (TGA) curves of the GNP nanocomposites, while Table 4 presents the onset of decomposition temperature (T_onset_) and maximum degradation temperature (T_max_). T_max_ was obtained from the DTG curves. T_onset_ represents the temperature at which significant thermal degradation begins. The neat blend (GNCS-0) has the lowest T_onset_ (386.38 °C), indicating lower thermal stability due to the absence of GNP reinforcement. The incorporation of 1 phr GNPs (GNCS-1) significantly increases T_onset_ to 398.08 °C, suggesting that GNPs enhance thermal resistance by acting as a barrier against thermal degradation. However, at higher GNP loadings (GNCS-4), T_onset_ decreases to 383.89 °C, likely due to GNP agglomeration, which creates structural inhomogeneities and reduces the reinforcement efficiency.

The T_max_ values were obtained from the derivative thermogravimetric (DTG) curves (Table 4). The T_max_ values, representing the temperature at which the highest degradation rate occurs, show an initial increase with GNP incorporation, reaching a peak at GNCS-1 (430.06 °C) compared to GNCS-0 (410.38 °C). This improvement suggests that well-dispersed GNPs contribute to enhanced thermal stability by acting as a heat shield, reducing heat transfer within the matrix. However, at higher GNP loadings (GNCS-2 to GNCS-4), T_max_ remains relatively stable (~420 °C), indicating that beyond a certain concentration, additional GNPs do not significantly improve the thermal barrier effect. This plateauing effect can be attributed to increased nanoplatelet agglomeration, which reduces the available surface area for effective thermal shielding and may introduce localized stress points within the matrix. Similar trends have been observed in polymer nanocomposites, where an optimal filler concentration enhances thermal stability but excessive loading leads to diminishing returns [54]. These findings highlight that 1 phr GNPs provides the best balance between dispersion and reinforcement, contributing to the highest thermal stability.

Overall, the TGA and DTG results show that GNP incorporation enhances thermal stability, as evidenced by the higher T_onset_ and T_max_ values compared to the neat blend. The 1 phr GNP nanocomposite (GNCS-1) exhibits the best thermal performance, suggesting optimal dispersion and stronger interfacial interactions, which is also supported by the morphological results (Figure 2b). The thermal stability decreases at higher GNP loadings (GNCS-4), possibly due to agglomeration effects, which limit reinforcement efficiency, and the agglomeration of GNPs at 4 phr is clearly visible in the FESEM micrographs (Figure 2d). The TGA results are consistent with the results of previous thermoplastic nanocomposite studies [55].

### 3.6. Limiting Oxygen Index (LOI) of the HNT/GNP Hybrid Nanocomposites

The limiting oxygen index (LOI) test results for the RPET/PA-11/Joncryl^®^/GNP nanocomposites showed a gradual improvement in flame retardancy with the addition of GNPs. The LOI values were determined based on the flow rates of oxygen and nitrogen required to maintain combustion. The results are presented in Table 5.

The control sample (GNCS-0), consisting of the polymer blend without filler, had the lowest LOI of 19, with an oxygen flow rate of 60.5 mL/min and a nitrogen flow rate of 116.5 mL/min. The low LOI indicated poor flame resistance, as the sample burned easily under ambient oxygen conditions. When 1 phr GNPs (GNCS-1) was added, the LOI increased to 21, with oxygen and nitrogen flow rates of 66 mL/min and 113.5 mL/min, respectively. The sample began to burn after 30 s, but the flame did not completely consume the 2.5 cm sample within 180 s. The flame extinguished shortly after the burner was removed, indicating an improvement in flame resistance due to the presence of GNPs, which acted as a heat barrier.

For GNCS-2 (2 phr GNPs), the LOI was initially measured at 21 with oxygen and nitrogen flow rates of 66 mL/min and 113.5 mL/min, but the sample did not ignite within 30 s. When the oxygen flow rate was increased to 69 mL/min (LOI = 22), the sample ignited after 30 s and self-extinguished within 60 s. Further tests with oxygen flow rates of 71.5 mL/min (LOI = 23) showed that the sample ignited after 30 s and self-extinguished within 80 s, while an oxygen flow of 74.5 mL/min (LOI = 24) resulted in the ignition and full consumption of the 2.5 cm specimen within 120 s. An LOI of 24 was recorded for GNCS-3 (3 phr GNPs) and GNCS-4 (4 phr GNPs) with oxygen and nitrogen flow rates of 74.5 mL/min and 109 mL/min, respectively. The GNCS-3 sample was completely consumed in 180 s, while the GNCS-4 sample burned slightly faster and was completely consumed in 150 s. A subsequent test for GNCS-4 at 71.5 mL/min oxygen (LOI = 23) consumed the sample in 140 s, reflecting the variability in flame behaviour due to filler agglomeration at higher GNP loadings.

The LOI results showed that the addition of GNPs significantly increased the flame retardancy of the nanocomposites. The control sample (GNCS-0) exhibited poor flame resistance due to the lack of reinforcement, while the GNP-containing formulations showed higher LOI values, indicating improved thermal stability and reduced flammability. The increase in LOI values with GNP loading suggests improved flame retardancy due to the barrier effect of graphene nanoplatelets, which restricts oxygen diffusion and promotes char formation. While the TGA results provide insights into the thermal stability of the materials, the LOI directly measures their resistance to combustion in an oxygen-enriched environment. The small variations in T_max_ observed in TGA do not fully account for the significant enhancement in the LOI, as flame retardancy is also influenced by factors such as heat shielding effects, char residue formation, and reduced flammable gas release during decomposition. The higher LOI values at increased GNP loadings confirm that beyond thermal stability, GNPs contribute to enhanced flame retardancy through multiple synergistic mechanisms. The formation of a stable char layer contributed to the flame-retardant properties by acting as a barrier to heat and mass transfer during combustion, as supported by other researchers [47].

### 3.7. UL-94 Vertical Burning Test of the GNP Nanocomposites

The UL-94 vertical flammability test results for the RPET/PA-11/Joncryl^®^/GNP nanocomposites showed a significant improvement in flame retardancy with the addition of GNPs, which is consistent with the LOI and TGA results. The control sample (GNCS-0) had the worst flame retardancy. It ignited within 10 s and continued to burn for an extended period of time, observing a drip that ignited the cotton beneath. The lack of reinforcement and the low LOI value of 19 (Table 5) reflected the poor flame retardancy of this formulation.

The addition of 1 phr GNPs (GNCS-1) improved flame resistance as the sample ignited within 10 s but self-extinguished within 5 s after the flame was removed. Although dripping occurred and the cotton ignited, the improvement was attributed to the even distribution of the GNPs, which acted as a heat barrier and retarded flame propagation. This result correlated with the LOI value of 21 (Table 5), indicating improved flame retardancy compared to GNCS-0. For GNCS-2, the sample also ignited within 10 s and extinguished within 10 s of flame removal, with dripping and cotton ignition observed. The increased GNP content further delayed flame propagation, consistent with the LOI value of 22–23 (Table 5) and the TGA results (Figure 7 and Table 4), which showed delayed thermal decomposition and increased char formation. Researchers reported that GNPs act as a flame-retardant filler in various polymer matrices [47,48,56].

GNCS-3 demonstrated better flame resistance, with ignition occurring within 7–8 s and extinguishing in the same period of time after flame removal. Although dripping and cotton ignition were still present, the shorter burning time demonstrated the effective heat barrier properties of GNPs at this loading. However, at 4 phr GNPs (GNCS-4), despite the higher filler content, the burn time was again 10 s, similar to GNCS-2. This behaviour suggests that the agglomeration of GNPs, as observed in the morphological analysis (Figure 2d), reduces their efficiency in improving flame retardancy at higher loadings. While GNCS-4 showed improved performance compared to the control, persistent dripping and cotton inflammation limited its classification as a flame retardant. The UL-94 test results confirm that the incorporation of GNPs enhances the flame retardancy of the RPET/PA-11 nanocomposites. The improved ratings observed at higher GNP loadings are attributed to the formation of a stable char layer, which acts as a thermal barrier and reduces the release of flammable volatiles. This behaviour aligns with the LOI findings, further supporting the role of GNPs in improving the flame resistance of the nanocomposites. However, the presence of dripping and cotton wool ignition in all the samples suggested that although GNP addition improved flame resistance, it was not enough to completely prevent burning dripping. This suggests that further optimization, such as combining GNPs with additional flame retardants, may be required to achieve higher classifications (e.g., V-0). The overall results are summarized in Table 6.

## 4. Conclusions

This study successfully developed new RPET/PA-11/Joncryl^®^/GNP nanocomposites by melt blending and comprehensively analysed their thermal, flame retardant, and mechanical properties. The key findings are as follows:

- The incorporation of 1 phr GNPs (GNCS-1) resulted in a well-balanced combination of tensile strength, flexural strength, and impact resistance. Higher GNP loadings (≥3 phr) led to increased stiffness but also promoted agglomeration, negatively affecting tensile and impact properties. Young’s modulus increased from 785.75 ± 171.5 MPa (GNCS-0) to 1210.6 ± 183.3 MPa (GNCS-2), while the flexural modulus improved from 3090.4 ± 273.4 MPa (GNCS-0) to 4170 ± 217.79 MPa (GNCS-4).
- The DSC analysis revealed that GNPs acted as a nucleating agent, influencing the cold crystallization behaviour of the blend. The crystallization temperature varied non-linearly with the GNP content, indicating complex filler–polymer interactions. The crystallinity (*X_c_*) increased from 4.19% (GNCS-0) to 25.73% (GNCS-1) and 31.28% (GNCS-2), confirming the nucleating effect of GNPs. The TGA results showed a moderate improvement in thermal stability, with T_onset_ increasing from 386 °C (GNCS-0) to 398 °C (GNCS-1) and T_max_ from 410 °C to 430 °C.
- The LOI results confirmed the flame-retardant role of GNPs, with values increasing from 19 (GNCS-0) to 23 (GNCS-1) and 24 (GNCS-2). However, at higher GNP loadings (≥3 phr), agglomeration limited further improvements in flame resistance. The UL-94 results further demonstrated the barrier effect of GNPs in improving flame retardancy.
- The FESEM analysis showed that 1 phr GNPs (GNCS-1) improved the phase dispersion of PA-11 within the RPET matrix. However, at higher concentrations (≥3 phr), agglomeration was evident, reducing the reinforcing efficiency.
- The FTIR spectra indicated that GNPs did not induce chemical modifications but influenced hydrogen bonding interactions within the polymer matrix. The peak intensity variations in the -OH and -NH stretching regions suggested improved interfacial adhesion.

Future Recommendations

To further optimize the performance and applicability of RPET/PA-11/GNP nanocomposites, the following aspects should be explored:

- Investigating finer increments (e.g., 0.5 phr steps) to determine the precise dispersion threshold.
- Exploring combinations of GNPs with other fillers to enhance mechanical, thermal, and flammability properties.
- Examining the electrical performance of RPET/PA-11/GNP nanocomposites, particularly for EMI shielding and electronic applications.
- Using surface functionalization or alternative processing techniques to minimize agglomeration.
- Evaluating melt processing behaviour for better control of flow properties during manufacturing.
- Assessing long-term stability under real-world environmental conditions to validate industrial applicability.

This study highlights the role of GNPs in enhancing the sustainability and performance of RPET/PA-11 nanocomposites, contributing to eco-friendly solutions for industries, such as automotive, packaging, and construction, while addressing plastic waste management challenges.

## 5. Significance of This Study

The automotive industry represents one of the largest markets for polymers due to their lightweight, durable, and versatile properties. Polymers such as PP, PU, PA6, PA-11, PA12, TPE, PE, PLA, and thermoplastic polyolefin (TPO) foams are widely used in manufacturing various automotive components, including bumpers, fender liners, body side mouldings, air dams, rocker panel covers, and wheel well mouldings. They also serve in critical engine compartment parts like air ducts, hood seals, and firewall pads, as well as interior elements such as airbag covers, dashboards, door panels, and instrument panels [57]. The tensile strength requirements for these components typically range from 18 to 50 MPa, a range achievable with the developed RPET/PA-11/GNP nanocomposites, making them suitable candidates for automotive applications [58,59].

The nanocomposites developed in this study not only meet these mechanical requirements but also offer improved thermal stability and flame resistance, which are critical for components exposed to high temperatures and potential fire hazards. Their enhanced rheological properties enable efficient processing techniques, such as injection moulding and extrusion, making them economically viable for large-scale production. Additionally, these materials are cost-effective, recyclable, and environmentally friendly, addressing the growing demand for sustainable solutions in the automotive industry.

Beyond automotive applications, the versatility of these nanocomposites extends to packaging, electronics, and electrical components, where lightweight, durable, and thermally stable materials are in high demand. They could be used in products such as laptop casings, printers, keyboards, and various moulded parts. By offering a sustainable solution for reusing PET waste, this research contributes to reducing plastic pollution while expanding the utility of recycled materials in industries requiring high-performance polymer composites. The developed nanocomposites thus provide a pathway to sustainable innovation across multiple sectors, promoting a circular economy and reducing environmental impact.

## Figures and Tables

**Table 1:** Formulation of the RPET/PA-11/Joncryl^®^/GNP nanocomposites.

Formulation (%)	RPET/PA-11 (wt%)	Joncryl^®^ (phr)	GNPs (phr)
GNCS-0	80:20	2	0
GNCS-1	80:20	2	1
GNCS-2	80:20	2	2
GNCS-3	80:20	2	3
GNCS-4	80:20	2	4

**Table 2:** Mechanical properties of the GNP nanocomposites.

Formulations	Tensile Strength (MPa)	Tensile Strain (%)	Young’s Modulus (MPa)	Flexural Strength (MPa)	Flexural Strain (%)	Flexural Modulus (MPa)	Impact Strength (J/m)
GNCS-0	44.825 ± 8.31	4.1 ± 0.69	785.75 ± 171.5	62.9 ± 3.10	2.3 ± 0.18	3090.4 ± 273.4	225.12 ± 72.45
GNCS-1	37.85 ± 2.13	2.2 ± 0.12	1036.6 ± 159.9	64.78 ± 5.26	1.9 ± 0.24	3580 ± 237.06	221.0225 ± 52.08
GNCS-2	36.94 ± 1.21	2.14 ± 0.09	1210.6 ± 183.3	64.82 ± 2.23	1.9 ± 0.08	3590 ± 103.6	195.725 ± 11.63
GNCS-3	36.54 ± 1.93	2.14 ± 0.11	1165.2 ± 148.6	64.84 ± 4.77	1.78 ± 0.16	3820 ± 157.3	189.005 ± 6.78
GNCS-4	36.3 ± 0.66	2 ± 0.07	1137.5 ± 115.32	65.88 ± 4.02	1.62 ± 0.17	4170 ± 217.79	185.552 ± 20.53

**Table 3:** DSC results of the GNP nanocomposites.

Formulations	Glass Transition T_g_ (°C)	Cold Crystallization Temperature T_c_ (°C)	Melting of Cold Crystallization Temperature T_m1_ (°C)	Melting Temperature T_m2_ (°C)	Crystallinity *Xc* (%)
GNCS-0	64.45	113.19	189.24	253.53	4.19
GNCS-1	65.57	117.08	189.94	252.26	25.73
GNCS-2	64.52	108.41	188.90	253.66	31.28
GNCS-3	65.24	119.44	191.00	255.13	30.90
GNCS-4	66.41	118.08	191.28	255.14	27.19

**Table 4:** T_onset_ and T_max_ of the GNP nanocomposites.

Formulations	Onset of Decomposition Temperature T_onset_ (°C)	Maximum Degradation Temperature T_max_ (°C)
GNCS-0	386.38	410.38
GNCS-1	398.08	430.06
GNCS-2	390.48	420.47
GNCS-3	391.35	420.82
GNCS-4	383.89	420.01

**Table 5:** LOI Results for GNP Nanocomposites.

Sample	O_2_ Flow Rate (mL/min)	N_2_ Flow Rate (mL/min)	LOI	Burning Behaviour
GNCS-0	60.5	116.5	19	Burned easily; poor flame resistance.
GNCS-1	66.0	113.5	21	Ignited in 30 s; not fully consumed in 180 s.
GNCS-2	66.0	113.5	21	Did not ignite in 30 s.
	69.0	112.0	22	Ignited in 30 s; extinguished in 60 s.
	71.5	110.5	23	Ignited in 30 s; extinguished in 80 s.
	74.5	109.0	24	Ignited in 30 s; consumed fully in 120 s.
GNCS-3	74.5	109.0	24	Ignited in 30 s; consumed fully in 180 s.
GNCS-4	74.5	109.0	24	Ignited in 30 s; consumed fully in 150 s.
	71.5	110.5	23	Ignited in 30 s; consumed fully in 140 s.

**Table 6:** UL-94 results for the GNP nanocomposites.

Sample	Ignition Time (s)	Burning Duration After Flame Removal (s)	Dripping	Cotton Ignition	Classification
GNCS-0	10	Prolonged	Yes	Yes	Not Classified
GNCS-1	10	5	Yes	Yes	V-2
GNCS-2	10	10	Yes	Yes	V-2
GNCS-3	7–8	7–8	Yes	Yes	V-2
GNCS-4	10	10	Yes	Yes	V-2

## Data Availability

Currently, the raw data that are critical to reproducing these results are not available for public sharing, as they are an essential part of an ongoing doctoral project. The raw data will be confidential until the project is completed and published.
